# Comparison of the Effectiveness of Body Surface Area Estimation Formulas in Predicting the Risk of Death in Patients with Heart Failure

**DOI:** 10.3390/jcm13216625

**Published:** 2024-11-04

**Authors:** Małgorzata Piecuch, Maciej Chylak, Michał Górski, Jagoda Garbicz-Kata, Anna Szczyrba, Marta Buczkowska, Jolanta Malinowska-Borowska, Jolanta Urszula Nowak, Jacek T. Niedziela, Mariusz Gąsior, Piotr Rozentryt

**Affiliations:** 1 Department of Chronic Diseases and Civilization-Related Hazards, Faculty of Public Health in Bytom, Medical University of Silesia in Katowice, 40-055 Katowice, Poland; michal.gorski@sum.edu.pl (M.G.); jagoda.garbicz-kata@sum.edu.pl (J.G.-K.); anna.szczyrba.09@gmail.com (A.S.); mbuczkowska@sum.edu.pl (M.B.); jmalinowska@sum.edu.pl (J.M.-B.); prozentryt@sum.edu.pl (P.R.); 2 Cardiology Outpatients Clinic, John Paul II Child and Family Health Center in Sosnowiec Sp. z o.o., 41-218 Sosnowiec, Poland; mchylak@mp.pl; 3 3rd Department of Cardiology, Faculty of Medical Sciences in Zabrze, Medical University of Silesia in Katowice, 40-055 Katowice, Poland; jnowak@sum.edu.pl (J.U.N.); jniedziela@sum.edu.pl (J.T.N.); mgasior@sum.edu.pl (M.G.)

**Keywords:** body surface area, heart failure, risk of death, obesity paradox

## Abstract

**Background/Objectives**: Body surface area is one of the most important anthropometric parameters in medicine. The study’s primary objective is to compare the consistency of the BSA estimation results through applying available formulas. Other objectives include determining the ability of these formulas to discriminate between death and survival in patients, comparing the formulas’ diagnostic features, and investigating whether the risk associated with a low BSA is independent of BMI. **Methods**: This study included 1029 patients (median age, 54 years; female, 13.7%; NYHA I/II/III/IV, 6.3%/36.5%/47.7%/9.5%) diagnosed with heart failure. For each patient, BSA was calculated using 25 formulas. Over the 3-year observation period, 31.2% of the patients died. **Results**: The average BSA value of the optimal discrimination thresholds was 1.79 m^2^ ± 0.084 m^2^ and the BSA difference between the estimators with the lowest (BSA_Meeh1879_) and the highest (BSA_Nwoye1989_) optimal discrimination thresholds was 0.42 m^2^. The lowest mortality rate was 35.2% and occurred in the subgroup of individuals with BSA values below the optimal discrimination threshold using the BSA_Schlich2010_ estimator. The highest mortality was predicted when the estimator BSA_Meeh1879_ or BSA_Livingston&Lee2001_ was used. **Conclusions**: Our study showed a relatively good concordance of 25 BSA estimators in BSA assessment in patients, without extremes of weight or height being known to disrupt it. All BSA estimators presented a significant, although weak, ability to discriminate death from survival at 3-year follow-up; however, BSA is not a very good predictor of HF mortality at 3 years. The higher risk of death in smaller patients, as shown by BSA, was independent of BMI in all but two BSA estimators.

## 1. Introduction

Body surface area (BSA) is one of the most important anthropometric parameters in modern medicine. Its direct measurement is difficult and requires sophisticated equipment [1]. In everyday clinical practice, different formulas are used to estimate body surface area. From the first formula published in Germany in 1879 to modern times, several dozen other formulas have been developed [2].

Although the basic anthropometric index remains the Quetelet index proposed in 1832, today known as the body mass index (BMI) [3], it is increasingly subject to criticism. One of the reasons for this criticism is its inability to distinguish the variable proportion of body fat in body composition [4]. Fat mass has a lower specific weight than muscle mass, and its increase takes up more space than an equivalent increase in muscle mass. This fact is more accurately reflected in changes in BSA than in BMI. For this reason, BSA measurement has gained importance in recent years as a potential better indicator of changes in body weight composition [5].

BSA measurement is used to normalize the results of a number of structural and functional studies, such as measured glomerular filtration, ultrasound examinations of the heart, and hemodynamic measurements [6,7,8]. Estimated BSA is also used in transplantology, burn treatment, and the appropriate selection of medication dosages in oncology and pediatrics [9,10,11,12].

Recently, there has been growing interest in using BSA to analyze the obesity paradox in chronic diseases. Studies indicate an association of lower BSA values with poorer prognosis. Such observations come from studies of patients with heart failure (HF) [13,14], acute kidney injury [15], and peripheral artery disease [16].

All the above-mentioned studies were conducted using the Mosteller formula from 1987 [17]. Although this formula is widely used, it is not the only one. Over the last 150 years, more than 20 different formulas have been published, but their validation is insufficient. Recently, inconsistencies in BSA estimates have been identified when using different formulas [2].

In contrast with the Mosteller formula, the association between poorer prognosis in chronic diseases and BSA estimated by other methods has not been documented. Although the justification for BSA research in the obesity paradox was the hypothesis that this parameter might outperform BMI, there are no convincing data supporting such a hypothesis.

The first objective of this study was to compare the consistency of BSA estimation results using the available formulas. The second aim was to determine the ability of these formulas to discriminate between death and survival in patients. The third objective was to compare the diagnostic features of the available formulas, and the last objective was to determine whether the risk associated with low BSA is independent of BMI.

## 2. Materials and Methods

This study was conducted using data from the Prospective Heart Failure Registry maintained at the Silesian Center for Heart Diseases in Zabrze in 2004–2014. It included patients diagnosed with HF who were assessed for indications for heart transplantation. At the time of inclusion in the registry, patients had circulatory control, no clinical signs of overhydration, and no peripheral edema. The registry protocol was approved by the Bioethics Committee of the Medical University of Silesia (NN-6501-12/I/04; date of approval, 12 January 2004), and the participants provided informed written consent to participate in the study. All procedures were undertaken in accordance with the Declaration of Helsinki.

A total of 1029 patients were included in the analysis, with a median age of 54 years, and 13.7% were women. The etiology of HF was ischemic in 62.9% of cases, while in the remaining patients, it had another origin. The median duration of HF was 35 months. The patients were on stable and optimal pharmacological treatment. Patients were classified into available treatment regimens based on the clinical criteria in effect at the time of registry maintenance. They were in different NYHA classes, and the percentages of patients in class I, II, III, and IV were 6.3%, 36.5%, 47.7%, and 9.5%, respectively. The median height was 1.73 m, and the median BMI of the entire group was 26.2 kg/m^2^. The minimum and maximum values of these parameters were 1.4 and 2.0 m and 40.0 and 139.7 kg, respectively. The percentage distribution of BMI categories according to the recommended classifications was 2.4%, 36.4%, 40.9%, 16.6%, 3.3%, and 0.4%, for the categories <18.5 kg/m^2^, 18.5–24.9 kg/m^2^, 25.0–29.9 kg/m^2^, 30.0–34.9 kg/m^2^, 35.0–39.9 kg/m^2^, and >40 kg/m^2^, respectively.

Patients were treated according to the guidelines applicable during the registry period. The percentages of patients receiving ACE inhibitors/ACE receptor antagonists, β-blockers, aldosterone antagonists, and loop diuretics were 93.1%, 97.6%, 92.2%, and 87.2%, respectively. Over the 3-year observation period, 31.2% of the patients died.

### Statistical Analysis

Continuous data were presented as medians and interquartile ranges, while categorical data were presented as percentages. For each patient, BSA was calculated using the 25 formulas presented in Table 1. For all the BSA calculation formulas used, an analysis was conducted to assess their ability to discriminate between the deceased and the living during a 3-year follow-up period. ROC analysis was used for this purpose, and the discriminatory ability of BSA calculated from the various formulas was presented in the form of areas under the ROC curve with 95% confidence intervals and *p*-values.

To determine the optimal discrimination value, the Youden method was used, and this value was calculated for each BSA formula. ANOVA or chi-square tests were used to compare the parameters characterizing the formulas. Pearson’s linear correlation analysis was used to examine the relationship between discrimination thresholds and patient numbers in subgroups, as well as the relationship between threshold values and mortality.

Next, the Kaplan–Meier survival curves were compared for BSA values < and ≥ the discrimination points for the BSA estimation methods with the lowest BSA (BSA_Meeh1879_) and highest (BSA_Nwoye1989_) discrimination points, as well as for the methods where the mortality below the discrimination points was the lowest (BSA_Schlich2010_) and the highest (BSA_Meeh1879_) [2]. Survival curves were compared with the use of the log-rank test.

In the next step, the Cox proportional hazards method was used to examine the relationship between lower BSA values and the identified optimal thresholds after taking into account the influence of confounding factors, primarily BMI.

In the final stage, for each of the 25 BSA estimation formulas, the chi-square test was used in the pairwise comparison of the percentiles of patients in subgroups with BSA values lower than the optimal threshold for that formula with the percentiles of BSA in subgroups below the optimal value for the recommended BSA estimator BSA_Mosteller1987_ from the literature. The mortality rate of patients was also compared between the resulting pairs. In all analyses, a *p* value of <0.05 was considered statistically significant. Multiple imputation techniques were used to fill in missing data (generally <5%).

## 3. Results

### 3.1. BSA and Prognosis

Regardless of the formula used for calculations, BSA was found to be a significant, though weak, predictor of mortality risk. The mean area under the ROC curve was 0.552 ± 0.0015, with the lowest area being 0.549 (BSA_Nwoye1989_) and the highest being 0.556 for the two estimators BSA_Choi1956_ and BSA_Schlich2010_. The coefficient of variation of the areas under the ROC curves was only 0.29% (Table 1).

In the ROC analysis, thresholds that optimally discriminated between living and dead patients were identified. These values differed significantly between the BSA estimation formulas.

**Figure 1:**
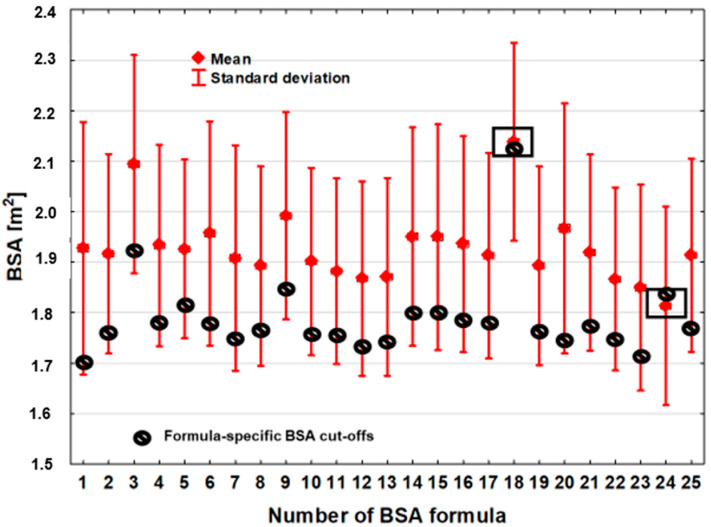
Comparison of BSA values estimated from 25 formulas (means and standard deviations in red) and optimal discrimination values for individual formulas (black circles).

The average BSA value of the optimal discrimination thresholds was 1.79 m^2^ ± 0.084 m^2^, and the BSA difference between the estimators with the lowest (BSA_Meeh1879_) and the highest (BSA_Nwoye1989_) optimal discrimination thresholds was 0.42 m^2^ (Table 2, Figure 1). The variation in BSA threshold values that optimally differentiated the deceased from the living was also slightly greater compared to the BSA estimation. The coefficient of variation (CV) was 4.7%.

**Figure 2:**
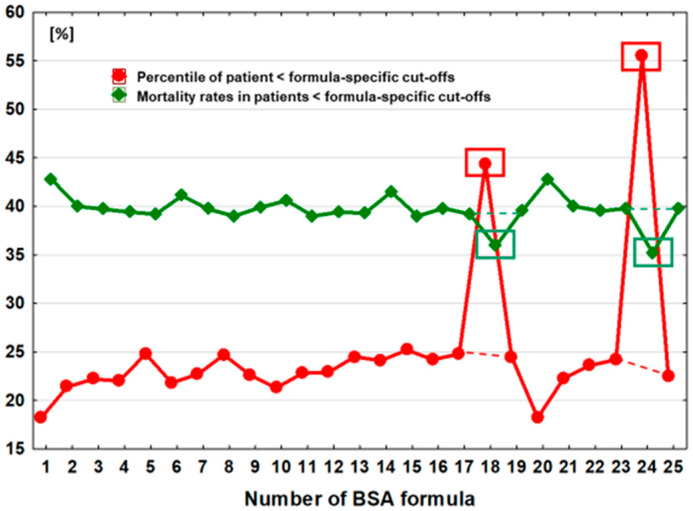
Percentiles of patients with BSA values estimated by individual formulas < optimal discrimination thresholds and mortality in these groups. Red rectangles indicate two formulas for which values < threshold are not an independent risk factor for death after adjusting for age, sex, and BMI (excluded from the analysis). Green rectangles indicate mortality related to these subgroups, which were excluded from the analyses.

The number (percentile) of subgroups with BSA values below the optimal discrimination value was strongly associated with the values of the optimal discrimination thresholds for the individual formulas (r = 0.59, *p* = 0.002) and ranged from 18.2% of the entire studied cohort, observed in the case of the two estimators BSA_Meeh1879_ and BSA_Livingston&Lee2001_, to 55.5%, in the case of BSA calculations made by using the BSA_Schlich2010_ formula (Table 2, Figure 2).

The difference in BSA values in terms of optimally discriminating prognosis in this case ranged from 0.10 to 0.14 m^2^, depending on the choice of the BSA_Meeh1879_ or BSA_Livingston&Lee2001_ formula. The coefficient of variation (CV) for the subgroup sizes was as high as 31.5%.

Mortality was negatively correlated with the height of the optimal discrimination threshold (r = −0.57, *p* = 0.003) and with the number of subgroups with lower discrimination threshold values (r = −0.86, *p* < 0.0001). The lowest mortality rate was 35.2% and occurred in the subgroup of individuals with BSA values below the optimal discrimination threshold using the BSA_Schlich2010_ estimator. This threshold was 1.84 m^2^, and the percentage of the entire population below this value was 55% (Table 2, Figure 2). The highest mortality was observed when the estimators BSA_Meeh1879_ or BSA_Livingston&Lee2001_ were used. In this case, the optimal death discrimination thresholds were 1.70 m^2^ and 1.74 m^2^, respectively, and among the 18.2% of patients who were below this value, the mortality rate was 42.8% (Table 2, Figure 2). The coefficient of variation (CV) for mortality in these highlighted subgroups was 4.1%.

The cumulative probability of survival, analyzed by the Kaplan–Maier method in the BMI and BSA groups defined by dichotomous division according to the optimal discrimination value, showed a higher probability of death in patients with examined parameter values in the lower range. A poorer prognosis for patients in subgroups below these points was observed for both BMI and BSA, and in the case of BSA, it was independent of the specific estimator used.

**Figure 3:**
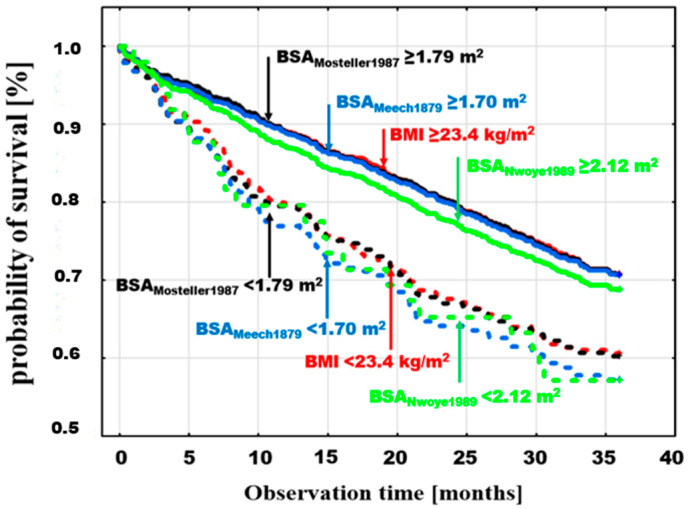
Cumulative probability of death in patients with BMI < 23.4 ≥ kg/m^2^ (log-rank *p* = 0.0008) with the BSA estimator with the lowest value of the optimal death discrimination threshold (BSA_Meeh1879_) < 1.7 ≥ m^2^ (log-rank *p* = 0.0001), the BSA estimator with the highest value of the optimal discrimination threshold (BSA_Nwoye1989_) < 2.12 ≥ m^2^ (log-rank *p* = 0.001), and BSA_Mosteller1987_, with thresholds < 1.79 ≥ m^2^ (log-rank *p* = 0.0001).

The differences between the probabilities of death for values < and ≥ optimal discrimination were significant for different BSA estimators. Example death probability graphs are presented in Figure 3, selecting, for comparison, the following: BMI, with threshold values < 23.4 ≥ kg/m^2^ (log-rank *p* = 0.0008); the BSA_Mosteller1987_ estimator, which is the most frequently used in clinical trials due to its simplicity and validation [13,14,15], with threshold values > 1.79 ≥ m^2^ (log-rank *p* = 0.0001); and two BSA estimators with extreme values of optimal discrimination points, the lowest being BSA_Meeh1879_ < 1.7 ≥ m^2^ (log-rank *p* = 0.0001) and the highest being BSA_Nwoye1989_ > 2.12 ≥ m^2^ (log-rank *p* = 0.001) (Figure 3).

**Figure 4:**
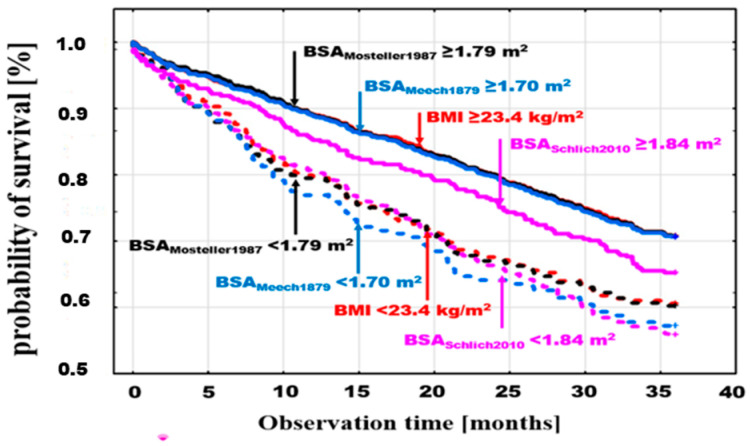
Cumulative probability of death in patients with BMI (log-rank *p* = 0.0008) and BSA_Mosteller1987_ (log-rank *p* = 0.0001) with the previously indicated thresholds and with BSA optimal discrimination with the highest mortality in the groups with low BSA values (BSA_Meeh1879_ < 1.7 ≥ m^2^ (log-rank *p* = 0.0001) and the lowest mortality (BSA_Schlich2010_) < 1.84 ≥ m^2^ (log-rank *p* = 0.006).

A similar comparison was performed by adding to BMI and BSA_Mosteller1987_ analysis the two estimators with the lowest and highest mortality in the groups of patients with BSA values below the optimal discrimination thresholds for these estimators: BSA_Meeh1879_, with a threshold of <1.7 ≥ m^2^ (log-rank *p* = 0.0001), and BSA_Schlich2010_, with a threshold of <1.84 ≥ m^2^ (log-rank *p* = 0.006), respectively (Figure 4).

**Figure 5:**
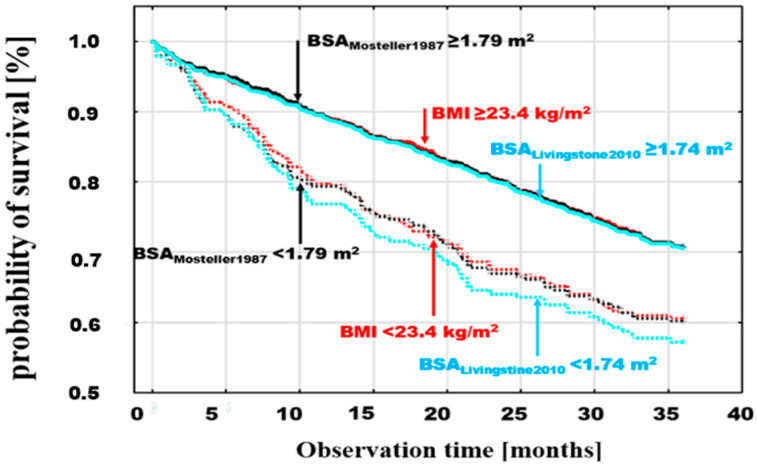
Comparison of survival curves for subgroups defined by the optimal discrimination values for distinguishing between the living and the deceased for the BSA_Mosteller1987_ and BSA_Livingston&Lee2001_ estimators < 1.74 m^2^ ≥ and for BMI.

In the final step, the survival curves for the dichotomous division of BMI and Mosteller with the thresholds indicated above were compared with the estimator that revealed the highest relative risk of death in the multivariate analysis (BSA_Livingston&Lee2001_), with the dichotomous division < 1.74 ≥ m^2^ (log-rank *p* = 0.0001) (Figure 5). The comparison of these estimators, with the thresholds indicated above, shows an overlap of the curves for patients above the respective thresholds (Figure 5). In contrast to these subgroups, patients with values below the thresholds had different risks. The risk was similar for BMI < 23.4 kg/m^2^ and BSA_Mosteller1987_ < 1.79 m^2^ but lower for patients with BSA_Livingston&Lee2001_ < 1.74 m^2^.

### 3.2. Risk of Death—Multivariate Analysis

Multivariate analysis of the relative risk of death for patients with BSA < the optimal discrimination points for individual estimators, relative to patients with BSA ≥ these values, after adjusting for age, gender, and BMI in models, as confounding variables, showed an increased risk of death of 41–80% for most estimators (Table 1). This effect was independent of age, gender, and BMI. Factors such as comorbidities, age, and gender were accounted for in the multivariate analysis based on clinical assumptions. For the BSA_Nwoye1989_ and BSA_Schlich2010_ estimators, values lower than those determined based on the optimal discrimination points of these formulas were not associated with a significantly increased risk.

A unit decrease in BSA was associated with a different change in the risk of death, depending on the estimator. For example, a 0.1 m^2^ BSA reduction increased the risk by 3.0% (95%CI: 1.2–4.8%) for the BSA_Meeh1879_ estimator, by 2.2%; (95%CI: 0.6–3.9%) for the BSA_Mosteller1987_ estimator, by 3.0% (95%CI: 1.2–4.8%) for the BSA_Livingston&Lee2001_ estimator, and by 2.0%; (95%CI: 0.3–3.7%) for the BSA_DuBois&DuBois1916_ estimator.

For most of the estimators, BMI was not a significant predictor of mortality risk in multivariate models. For five BSA estimators, BSA_Breitmann1932_, BSA_Sendroy&Cecchini1954_, BSA_Mattar1989_, BSA_Nwoye1989_, and BSA_Schlich2010_, BMI turned out to be independent from BSA as a predictor of prognosis in multivariate analysis. In all these cases, for each 1 kg/m2 decrease in BMI value, the risk increased by 3–4% (detailed data not presented).

Age and gender were also significant predictors of risk for most of the BSA estimators. For all BSA estimators, the increase in risk for each additional 5 years was approximately 13%. Male gender was associated with a 20–35% higher risk than female gender. In the case of the BSA_Takahira1925_, BSA_Choi1956_, BSA_Nwoye1989_, and BSA_Schlich2010_ estimators, gender was not significantly associated with prognosis.

Multivariate analysis revealed that the dichotomous division of BSA into values < and ≥ the BSA optimal discrimination values for different BSA estimators identified groups of patients with different risk. The highest relative risk after accounting the effects of gender, age, and BMI was identified by this method with the BSA_Livingston&Lee2001_ estimator, which documented an 80% increase in relative risk (Table 1). Compared to the most commonly used estimator, BSA_Mosteller1987_, the increase in relative risk was 24 percentage points. The lowest relative risk was characterized by the BSA_Sendroy&Cecchini1954_ estimator, where the increase in relative risk was 41% (95%CI: 5–91%); *p* = 0.02.

### 3.3. Variability of Parameters After Excluding Estimators in Multivariate Analysis

The variability of various parameters in the presented analyses of 25 BSA estimation formulas largely depended on two formula estimation results, which did not provide evidence of a relationship with prognosis. A reanalysis of these formulas revealed significantly different values for the coefficient of variation (CV).

In the case of BSA median variability, it decreased to 2.1%, area under the ROC curve to 0.22%, and BSA optimal discrimination values decreased to 2.7%. Similarly, the variability of the ratio of the BSA optimal discrimination to the average BSA value of the estimator used decreased by almost half, to 1.5%.

**Figure 6:**
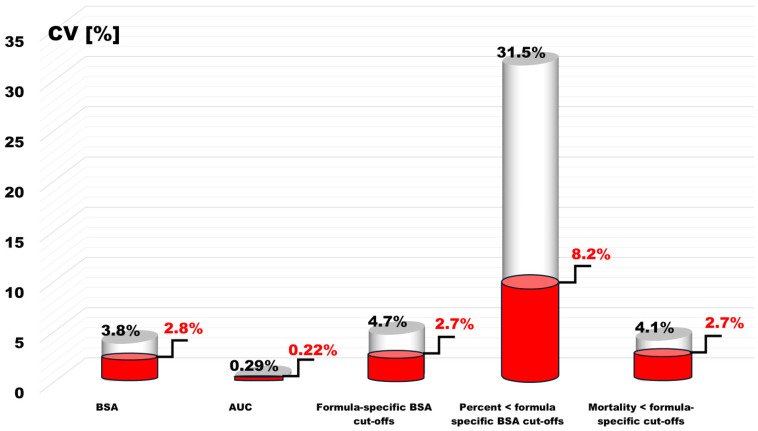
Comparison of the coefficients of variation (CVs) of all parameters characterizing the studied BSA estimation formulas. White—all 25 BSA estimators; red—with BSA_Nwoye1989_ and BSA_Schlich2010_ excluded.

As a result, there was a radical reduction, 8.2%, in the variability of the number of patients with BSA < optimal discrimination threshold and the variability in mortality within these groups, to reach 2.7% (Figure 6).

### 3.4. Percentiles and Mortality in Pairs < Formula-Specific Cut-Offs Versus < 1.79 m^2^ BSA_Mosteller1987_ Cut-Off

**Figure 7:**
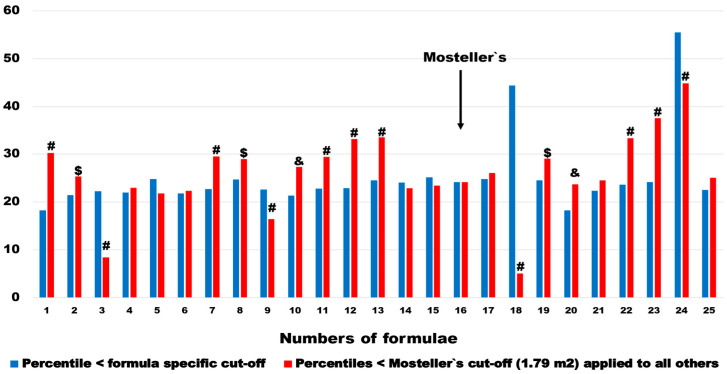
Pairwise comparison of numbers (percentiles) in subgroups of patients with BSA < the optimal discrimination threshold for a given formula and numbers below the threshold for the Mosteller estimator: &—*p* < 0.05, $—*p* < 0.01, #—*p* < 0.001.

The comparison of patient percentiles in pairs of formula-specific cut-offs and 1.79 m^2^ cut-off derived from the most recommended BSA_Mosteller1987_ formula shows huge differences. In 14 out of 24 BSA estimation methods (they were, by definition, equal in BSA_Mosteller1987_), these percentiles were significantly higher when the BSA_Mosteller1987_ cut-off was applied. In two estimators (BSA_Nwoye1989_ and BSA_Schlich2010_), which were further excluded from multivariable analysis, the mentioned percentiles were significantly lower (Figure 7). In the remaining eight estimators, there was no significant difference between pairs. The median change and interquartile range ware 2.5 and 7.9 percentage points, respectively.

**Figure 8:**
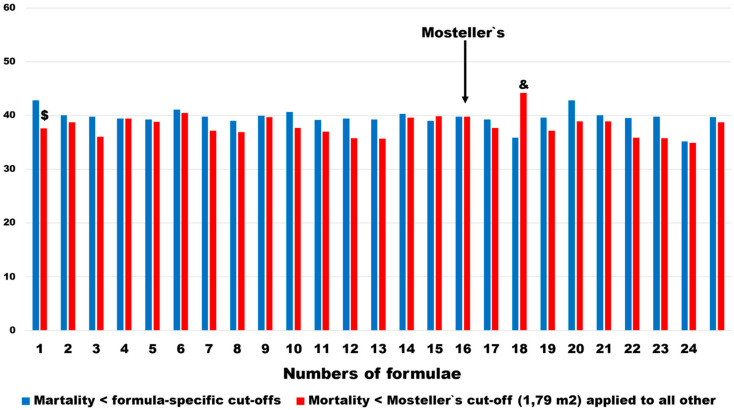
Pairwise comparison of patient mortality in subgroups with BSA < the optimal discrimination threshold for a given formula and numbers below the threshold for the Mosteller estimator: &—*p* < 0.05, $—*p* < 0.01.

The mortality rates changed less in pairs, as defined above. In all but one of the BSA estimating formulations, mortality rates were lower in subgroups defined as < BSA_Mosteller1987_ cut-offs. The difference was statistically significant only in BSA_Meeh1879_. In one BSA estimator (BSA_Nwoye1989_), which was further excluded from multivariable analysis, the mortality rates in patients with the < BSA_Mosteller1987_ cut-off were significantly higher (Figure 8). The median change and interquartile range of mortality risk were 1.5 and 3.3 percentage points, respectively.

## 4. Discussion

The phenomenon of better prognosis in overweight or mildly obese patients compared to normal weight patients in HF is known as an obesity paradox [18]. Despite more than 20 years of clinical and scientific research, its pathogenesis has remained largely elusive [19]. Numerous hypotheses have been put forward in order to explain this paradox, but none have reached common acceptance so far. Among the possible reasons for this failure, the use of inadequate obesity indices as a measure of risk has recently been suggested [20].

In spite of the widely known limitations of BMI in most studies, this anthropometric measure is still used for the categorization of body weight [4]. BSA is believed to overcome some obstacles linked to BMI. It may potentially better account for body composition variation and be more sensitive to central fat redistribution, because the higher muscle mass occupies less volume compared to the same mass of fat tissue. Hence, an increase in muscle mass impacts BMI more than BSA, and vice versa—an increase in fat tissue affects BSA more than BMI. A study shows that BSA is more closely correlated with numerous physiological parameters than BMI, can be successfully used for their indexing, and is linked to clinical outcome in HF [21].

In practice, BSA is not measured but is estimated by formulas. So far, at least 25 different formulations have been published [2]. The Mosteller formula, published in 1987 [17], has been used in studies on the obesity paradox in HF and found an association between lower BSA and worse clinical outcomes, not exceeding that noticed in the case of a lower BMI [13,14]. The Mosteller formula has been validated with other formulas only in terms of concordance in BSA estimation [22]. However, even perfect concordance in BSA estimation between formulas does not guarantee the same effects on prognosis. In each BSA estimation formula, the use of specific coefficients to adjust the key determinant of BSA for height and weight [23] may cause different representation of the central versus peripheral components of the body in the final BSA estimation. So far, no study has directly compared the prognostic performance of different BSA formulation in HF. Further, it is also unclear whether clinical outcome is more strongly correlated with BSA itself or with the structure of formulas, for example, promoting a larger representation of health-protecting peripheral portions of the body.

Our study shows a clinically meaningful discordance between certain formulations in the estimation of BSA among patients with HF. The extreme discordance found between BSA_Nwoye1989_ and BSA_Schlich2010_ was as high as 0.34 m^2^. Both of these formulas were further found to provide BSA estimation without any significant association with mortality. Their exclusion decreased by an extreme difference between BSA_Faber&Melcher1921_ and BSA_Mosteller1987_ to 0.25 m^2^. Based on published data on HF and BSA estimation using the Mosteller method, this difference would be associated with a 25% risk difference. Our BSA data derived from both Mosteller and Faber and Melcher assessments suggests that a 0,25 m^2^ lower BSA would increase risk by only 6% (95%CI: 2–10%), *p* = 0.006, in the case of BSA estimation by the Mosteller algorithm and 7% (95%CI: 1–9%), *p* = 0.02, when using the Faber and Melcher formula.

Our much lower figures of risk, as compared to Zafrir et al. [14], may be at least partly explained by there being adjustment for BMI in our study but not in that of Zafrir et al. Additionally, unlike in their study, we did not account for clinical parameters in our adjusted analysis. Finally, the follow-up times in the studies were different: 1 year in the study by Zafrir et al. and 3 years in ours.

A lower BSA estimated by the Mosteller method has shown significantly worse prognosis, and not only in patients with HF. A similar association has recently been demonstrated in acute kidney disease [15] in patients with peripheral arterial disease [16], but not in elderly patients after transcatheter aortic valve implantation [24]. In all of these studies, groups were constructed by dividing the cohort into tertiles or quartiles, without the identification of optimal cut-offs. In doing so, authors found that < 1.67 m^2^ in female patients and <1.93 m^2^ in male patients in acute kidney diseases [15] and < 1.70 m^2^ in peripheral arterial diseases [16] were associated with a significantly elevated risk for all-cause mortality. The BSA cut-offs set at the bottom quartile or tertile represent, on average, 25–30% of patients who remain on the higher-risk BSA spectrum. Our finding of BSA_Mosteller1987_ = 1.79 m^2^ for optimally separating higher from lower risk left 24.2% in the higher-risk area, which is quite similar to previously published data.

Our study is novel in that no previous studies tested alternative BSA formulations for correlation with prognosis, nor identified the optimal cut-off for mortality. Our study is the first to show the predictive performance of all 25 available BSA formulations in comparison to BMI in the same cohort of patients with HF.

Based on receiver operating characteristics, we observed striking differences in formula-specific cut-offs. The mean value of all 25 formula-specific cut-offs was 1.79 ± 0.08 m^2^. However, after excluding two formulas, as mentioned above, the mean cut-off decreased to 1.77 ± 0.05 m^2^. The value of certain cut-offs is important, but it is also crucial to consider where the cut-off point falls within the entire BSA range, defined by a given formula, and therefore, how many patients remain in higher-risk areas. In our study of 25 formulas, the average percentile of patients within higher-risk BSA levels was 25% ± 8%. After excluding the two formulas, the figure dropped down to 22.8% ± 1.8%). Another novelty of our work is the estimation of formula-specific, age-specific, gender-specific, and, for the first time in the literature, BMI-adjusted relative risk of death at 3-year follow-up. These analyses provided evidence for the lack of interchangeability in available BSA formulas when used for prognostication. In case of two BSA estimators’ adjustment for age, gender, and BMI completely disrupted the association between lower BSA and risk of death. The data for these estimators were excluded from all analyses. In all but five BSA estimators (BSA_Breitmann1932_, BSA_Sendroy&Cechhini1954_, BSA_Mattar1989_, BSA_Nwoye1989_, and BSA_Schlich2010_), a lower BMI remained independent of BSA as a predictor of mortality. With the exception of four BSA formulas (BSA_Takahira1925_, BSA_Choi1956_, BSA_Nwoye1989_, and BSA_Schlich2010_), gender was a significant predictor of death independently of BSA. All these findings suggest a high complexity in the interaction between weight, height, and gender—likely due to differences in body composition, which are differently reflected in BSA formulas. This complexity can be seen in population of elderly patients with polymorbidity who underwent transcatheter aortic valve implantation. In these patients, BSA did not predict mortality, and BMI only did over a short follow-up [24].

Our data also suggested that the use of the BSA formulation published by Livingston and Lee [25] would help in the identification of patients with the highest risk related to low BSA. The Kaplan–Meier curves of subgroups dichotomized according to optimal cut-offs from BSA_Livingston&Lee2001_, BSA_Mosteller1987_, and BMI showed overlapping risk profiles in the subgroups above formula-specific cut-offs and diverging risk profiles in the subgroups below respective cut-offs. The Livingston formula identified the highest-risk population without any trade-off effects in the lower-risk group.

The mortality risk was relatively constant across lower BSA levels, as identified by formula-specific respective cut-offs. If we consider the fact that in the two BSA estimators with the lowest mortality (BSA_Nwoye1989_ and BSA_Schlich2010_), multivariable analysis did not agree with a significant prognostic role for BSA below formula-specific cut-offs, then, in the remaining 23 formulas, in low-BSA groups, the mortality varied between 39% and 42.8%. This 3.8% mortality risk change occurred when formula-specific cut-offs differed by 0.23 m^2^, from 1.70 m^2^ to 1.93 m^2^. However, this difference occurred between the cut-offs of different BSA estimators. Yet, the increase in adjusted risk by 2–3% per 0.1 m^2^ decrease in BSA, as shown for BSA changes assessed by the same formula, cannot explain whether this mortality difference reflected inherent features of BSA formulations or was a consequence of lower BSA. The huge variation in the number of patients with subgroups identified based on different formula-specific cut-offs may speak in favor of the former explanation. Bearing in mind recent publications casting doubts on the obesity paradox as a true biological phenomenon, the weak association of BSA with mortality may be easier to understand.

## 5. Conclusions

In conclusion, our study has shown relatively good concordance among 25 BSA estimators in BSA assessment in patients, without extremes of weight or height known to disrupt concordance. Further, all BSA estimators presented a significant, although weak, ability to discriminate death from survival at 3-year follow-up. However, the area under the receiver operating characteristics (AUC) is very poor across all BSA indices, which indicates that BSA is not a very good predictor of HF mortality at 3 years. The formula-specific cut-offs identified based on ROC analysis allowed the separation of patients with a risk of death that was higher but relatively similar across different BSA formulas. However, a similar risk of death was, in a formula-specific manner, identified in highly variable fractions of patients. The higher risk of death in smaller patients as shown by BSA was independent of BMI for all but two BSA estimators. We found no justification for preferring the BSA_Mosteller1987_ formula in the study of risk associated with smaller body size. The BSA_Livingston&Lee2001_ estimator may offer better diagnostic performance.

### 5.1. Practical Relevance

Our findings may serve as a useful reference for clinicians searching for the best formula in the study of the obesity paradox.

### 5.2. Study Limitation

The main limitation of our study was the joint analysis of both female and male groups due to small number of females in the whole cohort. We attempted to correct this by adjusting for the difference in female patients in multivariable analysis. Other measures of obesity, such as the waist-to-hip ratio, may be better indicators than BMI, which was used in this study and could be worth including in future research studies. Selection bias, unmeasured and residual confounding, and the lack of external validation could be listed as other limitations of our study.

## Figures and Tables

**Table 1:** Selected BSA formulas [2].

Number	Author/s of the Formula	Formula
1	Meeh (1879)	0.1053 × W^2/3^
2	DuBois and DuBois (1916)	0.007184 × W^0.425^ × H^0.725^
3	Faber and Melcher (1921)	0.00785 × W^0.425^ × H^0.725^
4	Takahira (1925)	0.007246 × W^0.425^ × H^0.725^
5	Breitmann (1932)	0.0087 × (W + H) − 0.26
6	Boyd (1935)	0.0003207 × (W × 1000)^0.7285−0.0188 × log^_10_ ^(W × 1000)^ × H^0.3^
7	Stevenson (1937)	0.0128 × W + 0.0061 × H − 0.1529
8	Sendroy and Cecchini (1954)	0.0097 × (W + H) − 0.545
9	Banerjee and Sen (1955)	0.007466 × W^0.425^ × H^0.725^
10	Choi (1956)	men: 0.005902 × W^0.407^ × H^0.776^ women: 0.008692 × W^0.442^ × H^0.678^
11	Mehra (1958)	0.01131 × W^0.4092^ × H^0.6469^
12	Banerjee and Bhattacharya (1961)	0.007 × W^0.425^ × H^0.725^
13	Fujimoto et al. (1968)	0.008883 × W^0.444^ × H^0.42246^
14	Gehan and George (1970)	0.0235 × W^0.51456^ × H^0.42246^
15	Haycock et al. (1978)	0.024265 × W^0.5378^ × H^0.3964^
16	Mosteller (1987)	$\sqrt{(W\times H)/3600}$
17	Mattar (1989)	(W + H − 60)/100
18	Nwoye (1989)	0.001315 × W^0.262^ × H^1.2139^
19	Shuter and Aslani (2000)	0.0949 × W^0.441^ × H^0.655^
20	Livingston and Lee (2001)	0.1173 × W^0.6466^
21	Tikuisis (2001)	men: 0.01281 × W^0.44^ × H^0.6^ women: 0.01474 × W^0.47^ × H^0.55^
22	Nwoye and Al-Sheri (2003)	0.02036 × W^0.427^ × H^0.516^
23	Yu, Lo, Chiou (2003)	0.015925 × (W × H)^0.5^
24	Schlich (2010)	men: 0.000579479 × W^0.38^ × H^1.24^ women: 0.000975482 × W^0.46^ × H^1.08^
25	Yu, Lin, Yang (2010)	0.00713989 × W^0.404^ × H^0.7437^
26	Quetellet (1832)	W (in kg)/H (in meters)^2^

**Table 2:** Comparison of diagnostic efficiency of body surface area estimation formulas in discriminating deaths in patients with HF after 3 years.

	Author/s of the Formula	BSA or BMI (Median; Quartile Range) [m^2^/kg/m^2^]	Receiver Operating Characteristic Curve				Percentile of Patients < Formula-Specific Cut-Off [%]	Mortality < Formula-Specific Cut-Offs [%]	HR ± 95%CI < versus ≥ Formula-Specific Cut-Offs
			AUC	±95% Confidence Intervals	P for AUC	BSA or BMI Cut-Offs [m^2^/kg/m^2^]			
1	Meeh	1.92; (0.34)	0.553	0.515–0.591	0.006	1.70	18.2	42.8	1.51; (1.06–2.15), *p* = 0.02
2	DuBois and DuBois	1.92; (0.26)	0.551	0.515–0.589	0.008	1.76	21.4	40.0	1.48; (1.07–2.05), *p* = 0.02
3	Faber and Melcher	2.09; (0.29)	0.551	0.514–0.589	0.008	1.93	22.2	39.7	1.46; (1.06–2.01), *p* = 0.02
4	Takahira	1.93; (0.27)	0.551	0.514–0.589	0.008	1.78	22.0	39.4	1.43; (1.04–1.96), *p* = 0.03
5	Breitmann	1.92; (0.23)	0.551	0.513–0.589	0.008	1.81	24.8	39.2	1.44; (1.07–1.95), *p* = 0.02
6	Boyd	1.96; (0.30)	0.553	0.515–0.590	0.006	1.78	21.8	41.1	1.67; (1.19–2.30), *p* = 0.002
7	Stevenson	1.91; (0.29)	0.552	0.514–0.590	0.007	1.74	22.7	39.7	1.50; (1.09–2.07), *p* = 0.01
8	Sendroy and Cecchini	1.89; (0.26)	0.551	0.513–0.589	0.008	1.76	24.7	39.0	1.41; (1.05–1.91), *p* = 0.02
9	Banerjee and Sen	1.99; (0.27)	0.551	0.514–0.589	0.008	1.84	22.6	39.9	1.60; (1.09–2.06). *p* = 0.01
10	Choi	1.90; (0.25)	0.556	0.518–0.594	0.004	1.75	21.3	40.6	1.49; (1.09–2.03), *p* = 0.01
11	Mehra	1.88; (0.25)	0.551	0.514–0.589	0.007	1.75	22.8	39.0	1.44; (1.05–1.98), *p* = 0.03
12	Banerjee and Bhattacharya	1.87; (0.26)	0.551	0.514–0.589	0.008	1.73	22.9	39.4	1,46; (1.06–2.0), *p* = 0.02
13	Fujimoto et al.	1.87; (0.26)	0.552	0.514–0.589	0.007	1.74	24.5	39.3	1.47; (1.08–2.01), *p* = 0.01
14	Gehan and George	1.95; (0.29)	0.552	0.515–0.590	0.007	1.80	24.1	41.5	1.64; (1.19–2.25), *p* = 0.002
15	Haycock et al.	1.95; (0.30)	0.552	0.515–0.590	0.006	1.80	25.2	39.0	1.46; (1.06–2.00), *p* = 0.02
16	Mosteller	1.84; (0.29)	0.552	0.515–0.590	0.007	1.79	24.2	39.8	1.56; (1.14–2.14), *p* = 0.006
17	Mattar	1.91; (0.27)	0.551	0.513–0.589	0.008	1.78	24.8	39.2	1.44; (1.07–1.95), *p* = 0.02
18	Nwoye	2.15; (0.25)	0.549	0.511–0.587	0.01	2.12	44.4	35.9	1.20; (0.93–1.55), *p* = 0.15
19	Shuter and Aslani	1.89; (0.26)	0.552	0.514–0.589	0.007	1.76	24.5	39.5	1.51; (1.10–2.06), *p* = 0.01
20	Livingston and Lee	1.96; (0.34)	0.553	0.515–0.591	0.006	1.74	18.2	42.8	1.80; (1.28–2.55), *p* = 0.0007
21	Tikuisis	1.92; (0.26)	0.554	0.516–0.591	0.005	1.77	22.3	40.0	1.51; (1.10–2.08), *p* = 0.01
22	Nwoye and Al-Sheri	1.87; (0.24)	0.552	0.514–0.589	0.007	1.74	23.6	39.5	1.49; (1.09–2.05), *p* = 0.01
23	Yu, Lo, Chiou	1.85; (0.27)	0.552	0.515–0.590	0.007	1.71	24.2	39.8	1.56; (1.14–2.14), *p* = 0.006
24	Schlich	1.81; (0.26)	0.556	0.518–0.584	0.004	1.84	55.5	35.2	1.15; (0.89–1.50), *p* = 0.29
25	Yu, Lin, Yang	1.91; (0.25)	0.551	0.513–0.589	0.008	1.77	22.5	39.7	1.46; (1.07–2.00), *p* = 0.02
26	Quetellet (1832)	26.2; (30.8)	0.550	0.512–0.588	0.009	23.4	25.2	39.4	1.73; (1.36–2.199), *p* < 0.0001

Legend: BSA—body surface area, BMI—body mass index, AUC—area under receiver operating characteristics, HR—hazard ratio.

## Data Availability

Data are unavailable due to privacy.
